# Crossed Fused Renal Ectopia with Single Ureter and Single Renal Vein: A Rare Case

**DOI:** 10.7759/cureus.3914

**Published:** 2019-01-19

**Authors:** Sachin Khanduri, Ekta Tyagi, Vivek K Yadav, Sushma Pandey, Harsh Yadav, Mazhar Khan

**Affiliations:** 1 Radiology, Era's Lucknow Medical College and Hospital, Lucknow, IND

**Keywords:** cross, fused, renal ectopia with single renal vein, renal ectopia

## Abstract

Crossed fused renal ectopia with a single ureter and single renal vein is a rare congenital anomaly in which both the fused kidneys lie on one side of the body. We present an unusual case of crossed fused renal ectopia with a single ureter, single renal vein, and a calculus in a 22-year-old man who presented with lower abdominal pain, burning micturition, and a right-side abdominal lump present for three months. On abdominal ultrasound and non-contrast computed tomography (CT), the left kidney was not visualized in the left renal fossa. However, we noted it on the right side, fused to the lower pole of the right kidney with a calculus within the pelvis leading to mild hydronephrosis. After intravenous administration of non-ionic contrast medium, we saw a single ureter draining both the moieties into the urinary bladder on the right side. A three-dimensional volume rendering technique revealed a single renal vein draining the renal parenchyma into the inferior vena cava. Cross fused renal ectopia is an uncommon congenital anomaly which remains asymptomatic throughout life and undetected in the absence of renal pathologies. Multi-detector computed tomography (MDCT) is an excellent tool for denoting anatomical details of this anomaly; the information provided by MDCT is crucial for surgeons, nephrologists, and radiologists alike in facilitating proper management of the condition.

## Introduction

Crossed fused renal ectopia with a single ureter and single renal vein is a rare congenital anomaly in which both the fused kidneys lie on one side of the patient’s body. Crossed fused renal ectopia is usually drained by double pelvis and ureters which ultimately drain into the urinary bladder bilaterally. This condition is usually asymptomatic and found incidentally or when obstruction leads to episodic and radiating flank pain, burning micturition, episodic hematuria, and other constitutional symptoms (e.g., fever). We encountered an unusual case of crossed fused renal ectopia with single ureter, single renal vein, and a calculus. The blood supply to the kidneys in such cases shows many variations [1,2]. This variant of crossed fused renal ectopia has not been described in the literature to the best of our knowledge and is being followed up.

## Case presentation

**Figure 1:**
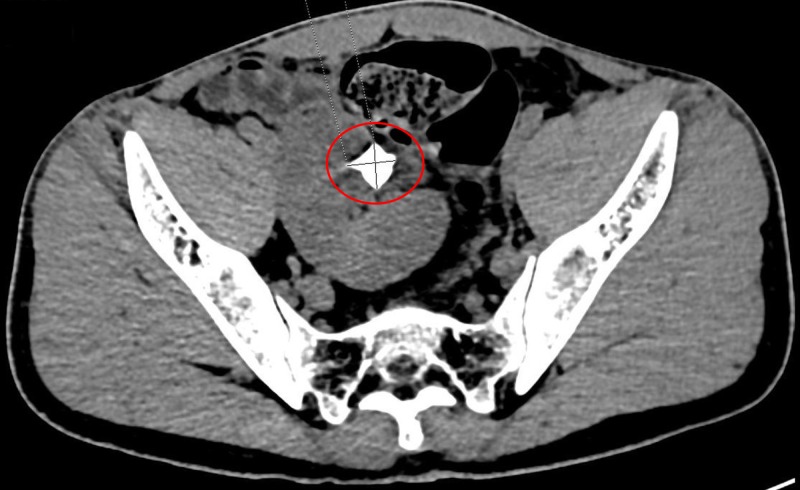
Non-contrast computed tomography of the abdomen revealing a left kidney on the right side fused to the lower pole of the right kidney.

A 22-year-old male patient presented with episodes of intermittent lower abdominal pain, burning micturition, and an abdominal lump in the lumbar region to the right of midline lasting for three months. The lump moved on inspiration and measured approximately 3.7 cm x 2.6 cm. The results of the patient’s renal function tests were within the reference range. An abdominal ultrasound (US) revealed a calculus measuring approximately 2 cm x 1.8 cm in the renal pelvis with obstructive features in the form of mild hydronephrosis on the right side. We did not see his left kidney in the left renal fossa. However, we noted a second kidney on the right side fused to the lower pole of the right kidney. Non-contrast computed tomography (CT) of the abdomen confirmed the US findings (Figure 1).

**Figure 2:**
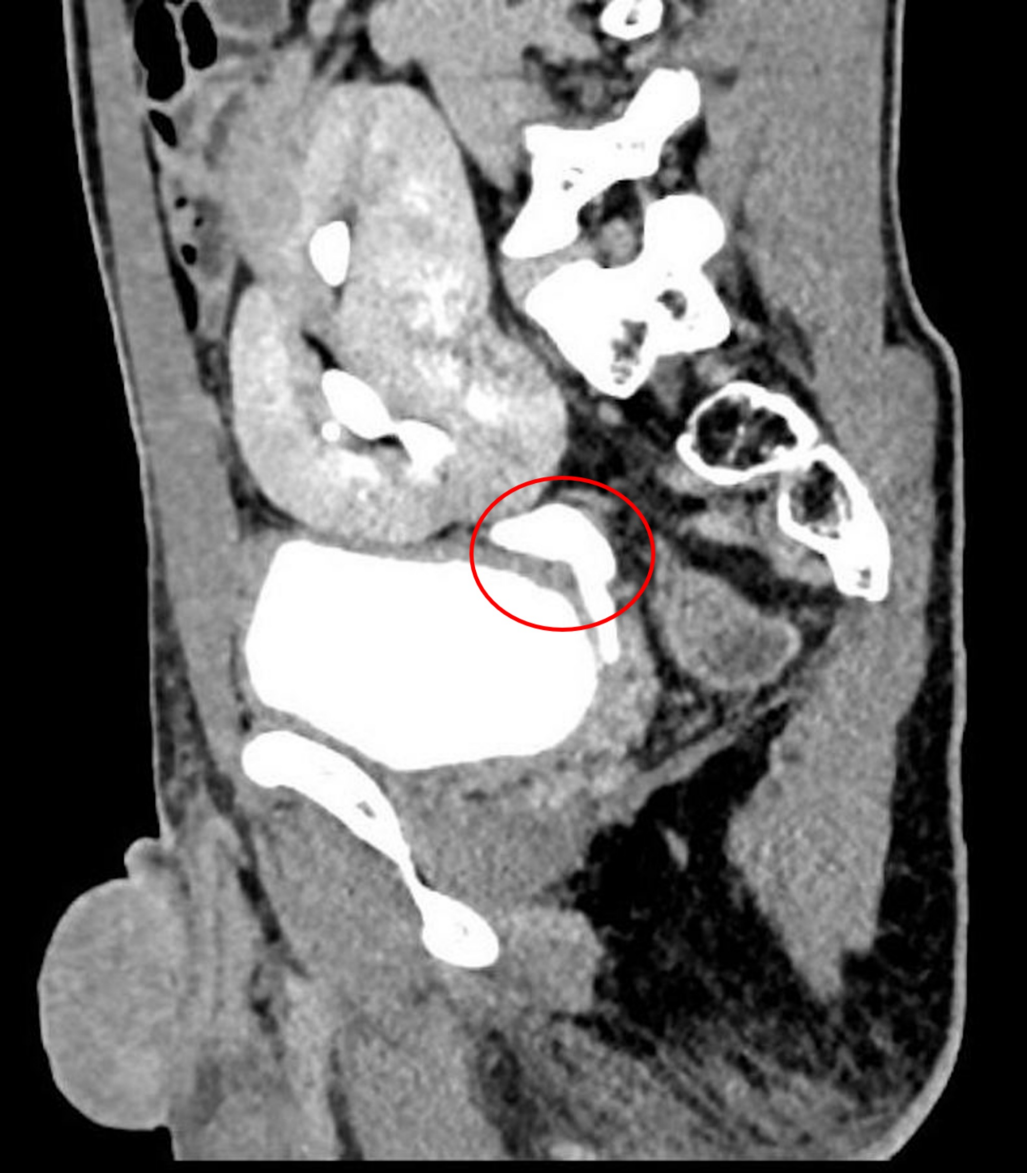
Computed tomography revealing a single ureter draining the collecting system of both the kidneys and terminally opening ipsilaterally into the urinary bladder.

On administration of intravenous non-ionic contrast agent, we noted a single ureter draining the collecting system of both the kidneys and terminally opening ipsilaterally into the urinary bladder (Figure 2).

**Figure 3:**
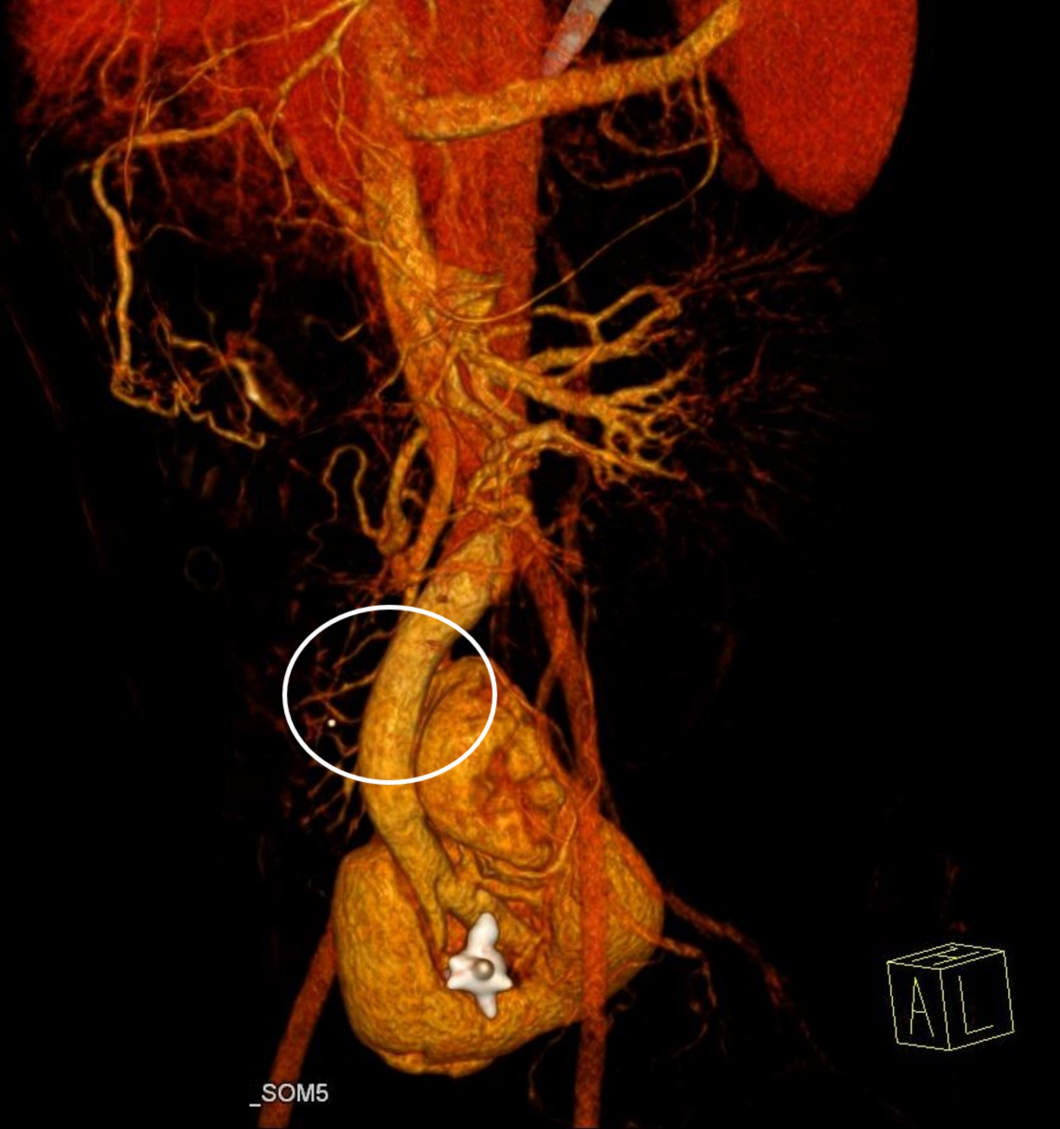
A three-dimensional volume-rendering revealed a single renal vein draining the fused renal parenchyma into the inferior vena cava.

**Figure 4:**
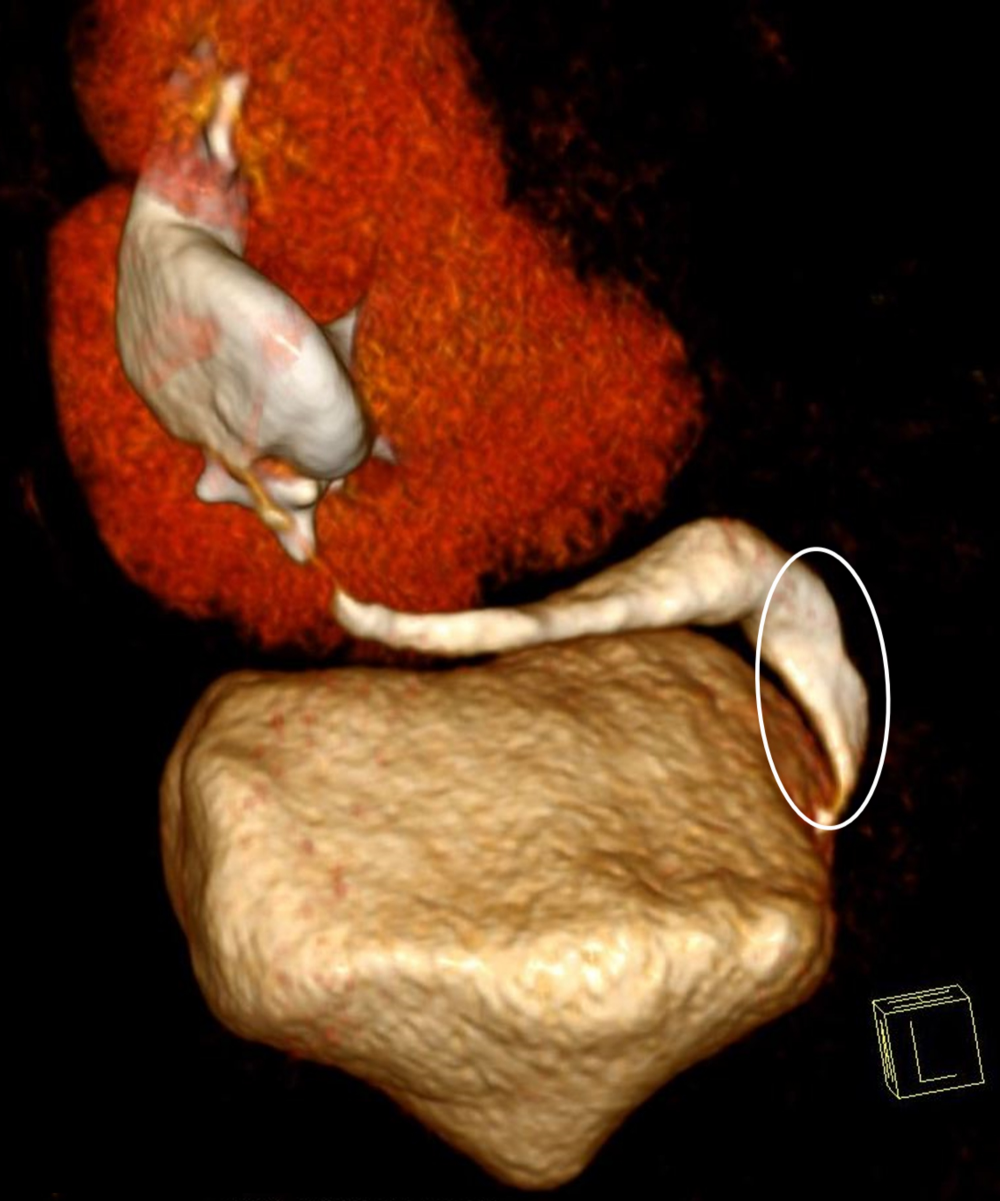
A three-dimensional volume-rendering revealed a single ureter draining the crossed fused kidneys into the urinary bladder on the same side.

However, the left ureter was absent. We noted a subtle thickening of the urinary bladder wall. The fused kidneys were supplied by two renal arteries originating from the left internal iliac artery. A three-dimensional volume-rendering technique revealed a single renal vein draining the fused renal parenchyma into the inferior vena cava (Figure 3) and a single ureter draining the crossed fused kidneys into the urinary bladder on the same side (Figure 4).

We saw no associated congenital skeletal abnormalities. However, the patient is under follow-up as such cases may show malignant transformation.

## Discussion

Crossed fused renal ectopia is the second most common congenital fusion abnormality (behind horseshoe kidney) with an incidence of approximately 1:1300 to 1:7500. In crossed fused renal ectopia, one of the kidneys crosses the midline and comes to lie on the opposite side and is fused to the inferior pole of the ipsilateral kidney. Crossed fused renal ectopia is usually drained by double renal pelvis and ureters which ultimately drain into the urinary bladder on both sides. The ureter of the ectopic kidney crosses the midline and enters the bladder on the opposite side [3]. Six variations of crossed fusion have been reported: type 1, inferior crossed fused ectopia; type 2, sigmoid or S-shaped kidney; type 3, unilateral lump kidney; type 4, unilateral disc kidney; type 5, L-shaped kidney; and type 6, superior crossed fused ectopia [4]. According to this classification system, two ureters drain both the kidneys separately into the urinary bladder at its orthotopic position. Crossed fused renal ectopia is rare due to presence of a single ureter and single renal vein. Most cases of renal ectopia remain asymptomatic throughout the patient’s lifetime and are diagnosed incidentally [5]. Patients usually present with intermittent episodes of lower abdominal or flank pain, burning micturition, episodes of hematuria, dysuria, and other constitutional symptoms like fever owing to infection in 30% of cases [6]. Nephrolithiasis, ureteropelvic junction obstruction, and hydronephrosis are associated with this condition [3,5-8]. Anomalies frequently associated with crossed ectopia are imperforate anus (4%), skeletal abnormalities (4%), and cardiovascular septal defects [3]. US is a good radiological modality to demonstrate the presence of fused renal ectopia. The sonographic findings usually reveal an absence of the kidney in the contralateral renal fossa or pelvis and fused kidneys on the ipsilateral side (with an anterior or posterior notch and different orientations of collecting systems) [9]. Contrast-enhanced CT helps make an accurate diagnosis of crossed fused renal ectopia with visualization of the number of draining ureters and its vascular supply for better surgical management. Renal cell carcinoma, transitional cell carcinoma, and Wilms’ tumor have been reported in crossed fused renal ectopia cases, which were managed by resection of the involved renal unit [10-13]. The embryological basis of crossed fused renal ectopia has not yet been clearly defined.

## Conclusions

Radiological modalities like US and multi-detector CT help in understanding the anatomy of the urinary system non-invasively by determining the number of ureters draining the crossed fused kidneys and its arterio-venous supply. Radiographic imagery further helps in planning the approach for surgical management in complicated cases.
